# Emission of cyanobacterial volatile organic compounds and their roles in blooms

**DOI:** 10.3389/fmicb.2023.1097712

**Published:** 2023-02-20

**Authors:** Zhaojiang Zuo

**Affiliations:** ^1^Zhejiang Provincial Key Laboratory of Forest Aromatic Plants-Based Healthcare Functions, Zhejiang A&F University, Hangzhou, China; ^2^State Key Laboratory of Subtropical Silviculture, Zhejiang A&F University, Hangzhou, China

**Keywords:** allelopathic effect, cyanobacterial blooms, repelling herbivore, transferring information, volatile organic compounds

## Abstract

Cyanobacteria are photosynthetic prokaryotes and one of dominant species in eutrophicated waters, which easily burst blooms in summer with high irradiance and temperature conditions. In response to high irradiance, high temperature, and nutrient conditions, cyanobacteria release abundant of volatile organic compounds (VOCs) by up-regulating related gene expression and oxidatively degrading β-carotene. These VOCs not only increase offensive odor in waters, but also transfer allelopathic signals to algae and aquatic plants, resulting in cyanobacteria dominating eutrophicated waters. Among these VOCs, β-cyclocitral, α-ionone, β-ionone, limonene, longifolene, and eucalyptol have been identified as the main allelopathic agents, which even directly kill algae by inducing programmed cell death (PCD). The VOCs released from cyanobacteria, especially the ruptured cells, exhibit repelling effects on the herbivores, which is beneficial to survival of the population. Cyanobacterial VOCs might transfer aggregating information among homogeneous species, so the acceptors initiate aggregation to resist the coming stresses. It can be speculated that the adverse conditions can promote VOC emission from cyanobacteria, which play important roles in cyanobacteria dominating eutrophicated waters and even bursting blooms.

## Introduction

Human activities lead to the continuous inputs of nutrients, especially nitrogen (N) and phosphorus (P), into inland waters, which aggravates the eutrophication (Peñuelas et al., 2013; Yang et al., 2020). Cyanobacteria are photosynthetic prokaryotes, which are quite different from eukaryotic algae. In eutrophicated waters, cyanobacteria massively multiply and grow, become the dominant species, and even burst blooms (Hudnell and Dortch, 2008). However, other aquatic organisms gradually reduce and even disappear at the end, including zooplankton, algae, aquatic plants, and fishes (Körner, 2001; Abrantes et al., 2006; Wu et al., 2019; Landsberg et al., 2020).

Cyanobacteria release a wide spectrum of volatile organic compounds (VOCs) through secondary metabolic pathways, mainly including terpenoids, sulfocompounds, benzenes, alkenes, furans, alkanes, alcohols, ketones, aldehydes, and esters (Walsh et al., 1998; Xu et al., 2017; Ye et al., 2018; Zuo et al., 2018a,b; Zuo, 2019). Their emission rate during cyanobacterial blooms can reach to approximately 81.2 μg·m^−2^·h^−1^ in Chaohu Lake in China (Liu M. et al., 2021), and the emission is sensitive to environmental conditions, such as nutrients (Xu et al., 2017; Ye et al., 2018; Zuo et al., 2018a,b), high irradiance (Walsh et al., 1998; Wang and Li, 2015; Zheng et al., 2020), and high temperature (Wang and Li, 2015; Zheng et al., 2020).

Cyanobacterial VOCs result in offensive odor in waters, of which geosmin, 2-methylisoborneol, β-cyclocitral, and β-ionone provide main contribution (Watson et al., 2016; Liu et al., 2017). For geosmin and 2-methylisoborneol, they are responsible for musty and earthy odors, and easily sensed by human, due to their extremely low odor thresholds (< 10 ng·L^−1^) (Watson et al., 2016). The toxicological effects of these odor compounds on human are still unknown, but they profoundly lower the water aesthetics and quality, and even cause drinking water crisis to more than two million residents for 5 days in Wuxi in China (Ma et al., 2013). Moreover, these VOCs serve important functions in cyanobacteria dominating eutrophicated waters, such as inhibiting and even killing algae and aquatic plants (Xu et al., 2017; Zuo et al., 2018b; Sun et al., 2020; Du et al., 2022), repelling herbivores (Zuo, 2019; Havaux, 2020; Saha and Fink, 2022), and possibly transferring aggregating information to induce the formation of blooms. In this review, the emission of VOCs from cyanobacteria and their functions in blooms are summarized to provide helps for uncovering cyanobacteria blooms from VOC perspective. To the best of my knowledge, this is the first specific review about the research advances of cyanobacterial VOCs.

## VOC emission in response to environmental conditions

### High irradiance

**Figure 1:**
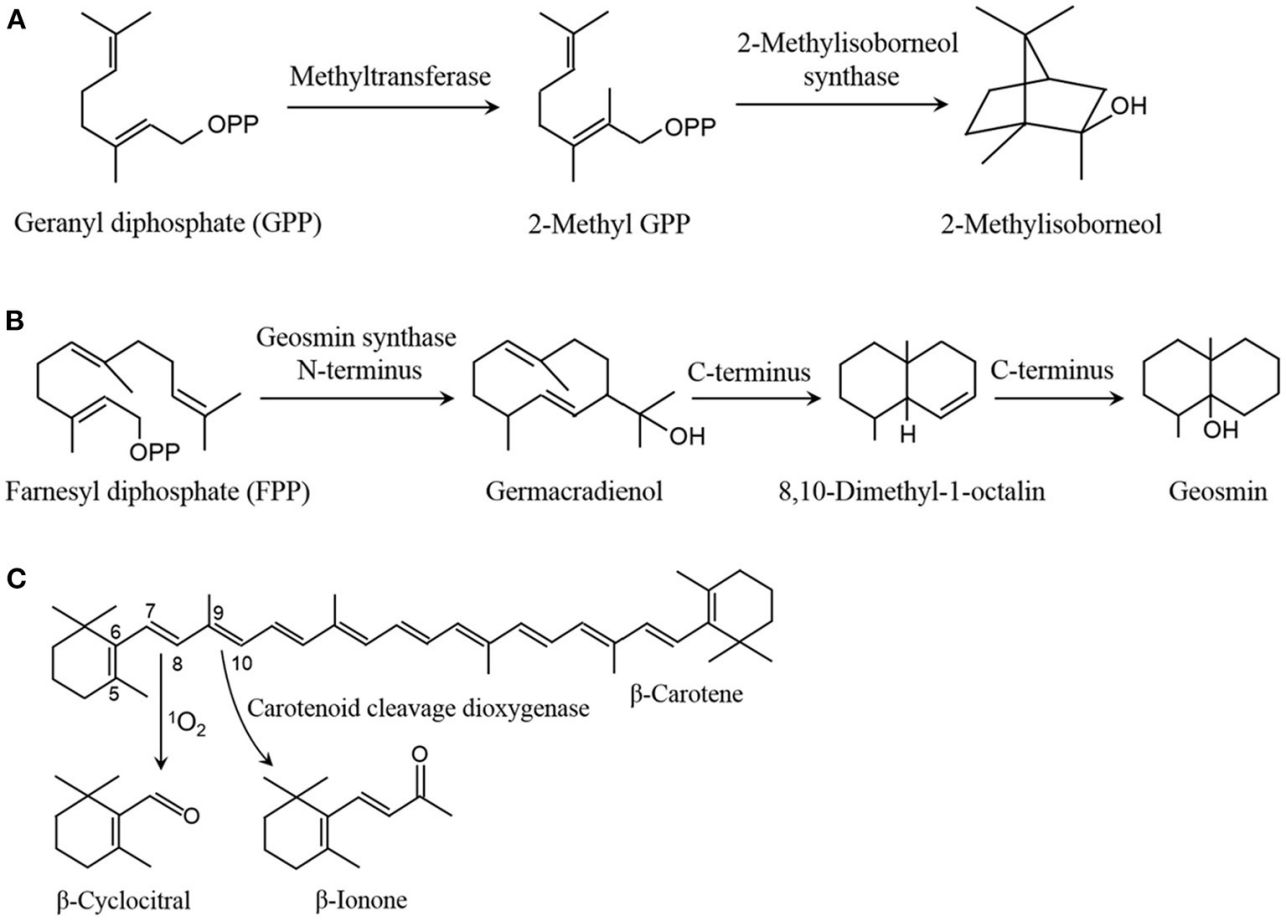
Pathway of 2-methylisoborneol **(A)**, geosmin **(B)**, β-cyclocitral and β-ionone **(C)** formation.

In eutrophicated waters, cyanobacteria massively grow in spring with suitable conditions, and they reach to the biomass peak and easily burst blooms in summer (June to August or September in the northern hemisphere) with high irradiance and temperature conditions (Beal et al., 2021). Under high irradiance, a remarkable improvement is detected in the emission amount of VOCs from cyanobacteria (Walsh et al., 1998; Wang and Li, 2015; Zheng et al., 2020). 2-Methylisoborneol and geosmin are two typical cyanobacterial VOCs, which can be released by over 70 cyanobacterial species, such as *Anabaena, Lyngbya, Nostoc, Phormidium, Oscillatoria, Aphanizomenon*, and *Planktothrix* (Izaguirre and Taylor, 2004; Watson et al., 2016). The emission of the two compounds varied with season and exhibited an increase trend from spring to late summer (Westerhoff et al., 2005), and their content can reach to 51.4 and 120.9 ng·L^−1^, respectively, in Eagle Creek Reservoir, Indiana (Clercin et al., 2022). 2-Methylisoborneol and geosmin belong to terpenoids, and are synthesized with geranyl diphosphate (GPP) from methylerythritol-4-phosphate pathway (MEP) and farnesyl diphosphate (FPP) from mevalonate pathway (MVA) as the precursor, respectively. In the formation of 2-methylisoborneol, GPP is transferred into a methyl from S-adenosylmethionine to form 2-methyl GPP, and then converted into 2-methylisoborneol by catalyzing with 2-methylisoborneol synthase (Figure 1A) (Giglio et al., 2011; Watson et al., 2016). In the generation of geosmin, FPP is transformed to germacradienol in the catalysis of geosmin synthase N-terminus, and then converted into geosmin through an intermediate compound 8,10-dimethyl-1-octalin by catalyzing with the enzyme C-terminus (Figure 1B) (Jiang et al., 2007; Watson et al., 2016). Compared with low irradiance, high irradiance obviously promoted the emission of 2-methylisoborneol from *Pseudanabaena* sp. and geosmin from *Anabaena ucrainica* (Wang and Li, 2015), which might be caused by up-regulating expression of the genes associated with the two compound biosynthesis (Devi et al., 2021).

β-Cyclocitral and β-ionone are another two typical compounds in cyanobacterial VOCs. Their emission amount can reach to a high level during cyanobacterial blooms in summer, e.g., β-cyclocitral and β-ionone were separately detected about 284.3 and 185.0 ng·L^−1^ in Lake Taihu, and about 538.12 and 50.44 ng·L^−1^ in Western Lake Chaohu, China (Ma et al., 2013; Jiang et al., 2016). The two compounds are derived from β-carotene degradation by oxidizing with reactive oxygen species (ROS), especially ^1^O_2_, and catalyzing with carotenoid cleavage dioxygenases (CCDs), respectively (Figure 1C) (Havaux, 2020; Zheng et al., 2020). There were three CCD homologs (NSCs) were identified in *Nostoc* sp. PCC 7120, of which two NSCs can cleave β-carotene at the C9=C10 double bonds to form β-ionone (Scherzinger et al., 2006). In cyanobacteria, no a CCD was found to be responsible for β-cyclocitral formation by cleaving β-carotene (Havaux, 2020; Zheng et al., 2020). Under high irradiance, an increase was found in the emission of β-cyclocitral and β-ionone from *Microcystis aeruginosa* (Walsh et al., 1998; Zheng et al., 2020). High irradiance elevated the ^1^O_2_ level in *M. aeruginosa*, which raised the emission of β-cyclocitral by oxidizing β-carotene at C7=C8 double bonds (Zheng et al., 2020).

### High temperature

High temperature, another typical environmental condition in summer, can also promote VOC emission from cyanobacteria (Wang and Li, 2015; Zheng et al., 2020). In contrast with 25°C, high temperature at 35°C promoted the emission of 2-methylisoborneol from *Pseudanabaena* sp. and geosmin from *A. ucrainica* (Wang and Li, 2015). This can be explained by up-regulation of the related genes in the two compound formation (Kakimoto et al., 2014; Watson et al., 2016). *Lobaria pulmonaria* is a symbiont of cyanobacterium *Nostoc*, green alga *Dyctiochloropsis* and fungus. Heat shock promoted the symbiont releasing carotenoid degradants as well as C6 green leaf volatiles (GLVs) that were derived from the oxidative degradation of fatty acids (García-Plazaola et al., 2017). Under the temperature at 25, 30, and 35°C, the emission amount of β-cyclocitral from *M. aeruginosa* gradually raised with elevating the temperature, due to the gradually increased ^1^O_2_ enhancing β-carotene oxidative degradation (Zheng et al., 2020).

### Nutrition conditions

There are multiple forms of N and P in eutrophicated waters, which have different effects on the VOC emission from cyanobacteria. *Microcystis aeruginosa* and *Microcystis flos-aquae* mainly released terpenoids, sulfocompounds, benzenes, furans, hydrocarbons, aldehydes, and esters. These VOC emission amount and compound types were quite different among nine N sources, with the maximum emission amount by using NH_4_Cl and maximum compound types by using urea (Xu et al., 2017; Zuo et al., 2018a). When the two *Microcystis* were supplied with sodium pyrophosphate, K_2_HPO_4_, and sodium hexametaphosphate as the sole P source, K_2_HPO_4_ and sodium hexametaphosphate separately promoted the VOC emission from *M. aeruginosa* and *M. flos-aquae* (Ye et al., 2018; Zuo et al., 2018b).

In water bodies, P is regarded as the limiting factor for cyanobacterial growth, of which concentration affects the VOC emission from cyanobacteria. The geosmin level in reservoirs was negatively related with the P concentration (Dzialowski et al., 2009). With declining the P content, the VOC emission amount from *M. aeruginosa* and *M. flos-aquae* gradually increased, and the highest emission amount was found under non-P condition (Ye et al., 2018; Zuo et al., 2018b). During 35-day cultivation, the emission amount of 2-methyl-1-butanol, 3-methyl-1-butanol, and 2-phenylethanol from *M. aeruginosa* gradually increased with exhausting the N nutrient, which might be caused by the raised activity of 2-keto-acid decarboxylase (Hasegawa et al., 2012). Similarly, *M. aeruginosa* and *M. flos-aquae* increased VOC emission with declining the N concentration, and the highest emission amount was detected under non-N condition, due to up-regulation of the genes in precursor formation of the VOCs, especially terpenoids and benzenes (Xu et al., 2017; Zuo et al., 2018a). These results indicate that the relative lack of nutrients resulted from cyanobacterial massive growth may promote the VOC emission. In addition, extremely high N level also improved VOC emission from *Microcystis*, due to the stress effects (Gan et al., 2015).

## Functions of VOCs in cyanobacterial blooms

### Allelopathic effects

When *M. aeruginosa* was co-cultured with a diatom (*Cyclotella meneghiniana*) and two green algae (*Scenedesmus quadricauda* and *Chlorella pyrenoidosa*), it showed allelopathic effects on the growth of algae (Wang et al., 2017). In field works, a positive relationship between cyanobacterial VOCs and the seasonal succession of algal species has been found, and *Microcystis* finally became the dominant species (Arii et al., 2021). When *Chlorella vulgaris* and *Chlamydomonas reinhardtii* were separately treated with the VOCs from *M. flos-aquae* and *M. aeruginosa* under non-N condition, the cell growth and photosynthetic properties declined remarkably (Xu et al., 2017; Zuo et al., 2018a). Similarly, the VOCs from *M. flos-aquae* and *M. aeruginosa* under non-P condition also exhibited inhibitory effects on the cell growth and photosynthetic properties in *C. vulgaris* and *C. reinhardtii* (Ye et al., 2018; Zuo et al., 2018b). Although there are plentiful nutrients in eutrophicated waters, the massive growth of cyanobacteria may result in the relative lack of nutrients. In that case, cyanobacteria release an abundance of VOCs to inhibit other competitors and promote themselves to dominant the waters (Zuo, 2019). In addition, high irradiance and temperature also exhibit promoting effects on the VOC emission, which should contribute to the allelopathic effects of cyanobacteria for offsetting the adverse effects of environmental conditions on cyanobacterial growth.

Among cyanobacterial VOCs, some components have been identified as the allelopathic agents, e.g., geosmin, β-cyclocitral, α-ionone, β-ionone, and geranylacetone exhibited inhibitory effects on the growth of *C. pyrenoidosa* (Ikawa et al., 2001). Limonene and eucalyptol were two main components in *Microcystis* VOCs, and can inhibit the growth of *C. vulgaris* and *C. reinhardtii* by degrading photosynthetic pigments and lowering photosynthetic properties (Zhao et al., 2016; Zhou et al., 2016). In addition, 0.4 mM β-cyclocitral, 0.2 mM α-ionone, 0.2 mM β-ionone, 0.2 mM limonene, 0.4 mM longifolene, and 1.2 mM eucalyptol even directly killed *C. reinhardtii* by inducing programmed cell death (PCD), with appearance of some typical characteristics, such as caspase-like activation, nuclei concentration firstly following by a broken process, and DNA ladders (Sun et al., 2020; Liu J. et al., 2021; Yin et al., 2021). In exposure to 0.1–0.5 mg·ml^−1^ β-cyclocitral, a rupture was found in *Nitzschia palea* cells (Chang et al., 2011). In addition, 21.2 mg·ml^−1^ (110.4 mM) β-ionone and 5 mg·ml^−1^ (32.9 mM) β-cyclocitral even poisoned their emitter *M. aeruginosa* (Chang et al., 2011; Shao et al., 2011). However, this extreme high concentration is hard to reach in natural waters, and algae have been killed in that case.

Besides algae, aquatic plants are also primary producers in aquatic ecosystems, and they massively decrease and even disappear during cyanobacterial blooms, leading to a macrophytic lake changing into an algal lake (Wu et al., 2019). In Lake Müggelsee in Germany, cyanobacterial blooms resulted in the reduction of aquatic plants for approximately 90% (Körner, 2001). When submerged plants were exposed to cyanobacterial exudates and extracts, their growth was impacted by increasing ROS levels and declining photosynthetic properties (Zheng et al., 2013; Zhang et al., 2021). Recently, the allelopathic effects of β-cyclocitral and β-ionone on duckweed (*Lemna turionifera*) were detected. The two compounds exhibited inhibitory effects on duckweed growth and photosynthetic properties by down-regulating expression of the genes related with photosynthetic pigment synthesis, photosynthetic electron transport chain, CO_2_ fixation, and growth-promoting hormone synthesis (Du et al., 2022). This indicates that cyanobacterial VOCs act as signal molecules to play allelopathic roles.

### Repelling herbivores

*Daphnia* (water fleas) are dominant herbivores in waters and primarily feed eukaryotic algae rather than cyanobacteria, as cyanobacteria contain more biochemical substances (Lürling, 2003; von Elert et al., 2003). When *Daphnia magna* was exposed to β-cyclocitral and 2,4,7-decatrienal, it exhibited an avoidance behavior by obviously increasing the swimming rate (Watson et al., 2007). Jüttner et al. (2010) reported that *Microcystis* cell rupture raised β-cyclocitral emission by activating a rapid β-carotene oxidation, and then the compound served repelling function to *D. magna*. This model is similar with damaged higher plants releasing VOCs to repel herbivores, but the repelling signal emission is beneficial to cyanobacterial population with sacrificing single celled individual. In addition, geosmin has also exhibited a repellent activity against herbivores (Saha and Fink, 2022).

### Transferring aggregating information

In terrestrial ecosystems, it has been well-studied in the VOC message transfer between plants (Baldwin et al., 2006; Loreto and D'Auria, 2022). This phenomenon has also been detected in green alga *C. reinhardtii*, indicating that it might be a retention mechanism during biological evolution (Zuo, 2019). The VOCs from *C. reinhardtii* undergoing PCD can induce health *C. reinhardtii* improving antioxidant enzyme activities for defense (Zuo et al., 2012a). The VOCs from *C. reinhardtii* stressed by NaCl and Na_2_CO_3_ also showed the similar inducing effects on health *C. reinhardtii* (Zuo et al., 2012b). During *C. reinhardtii* PCD triggered by wasp venom, the released NO and ethylene improved the tolerance of health *C. reinhardtii* cells to wasp venom (Yordanova et al., 2010). Cyanobacterial cells aggregate together and float onto the water surface to form blooms. This aggregation is important for enhancing cyanobacteria tolerance to the adverse environments, such as high irradiance, high temperature, nutrient deficiency, and herbivores (Ma et al., 2014; Zhu et al., 2014; Li et al., 2015). We have found that β-cyclocitral, limonene, longifolene, and eucalyptol can induce *M. aeruginosa* cell aggregation (unpublished data), indicating that the VOCs released under adverse conditions might transfer information to other cyanobacterial cells and induce them aggregating together (bursting blooms) to resist the coming stresses.

## Conclusion and prospection

**Figure 2:**
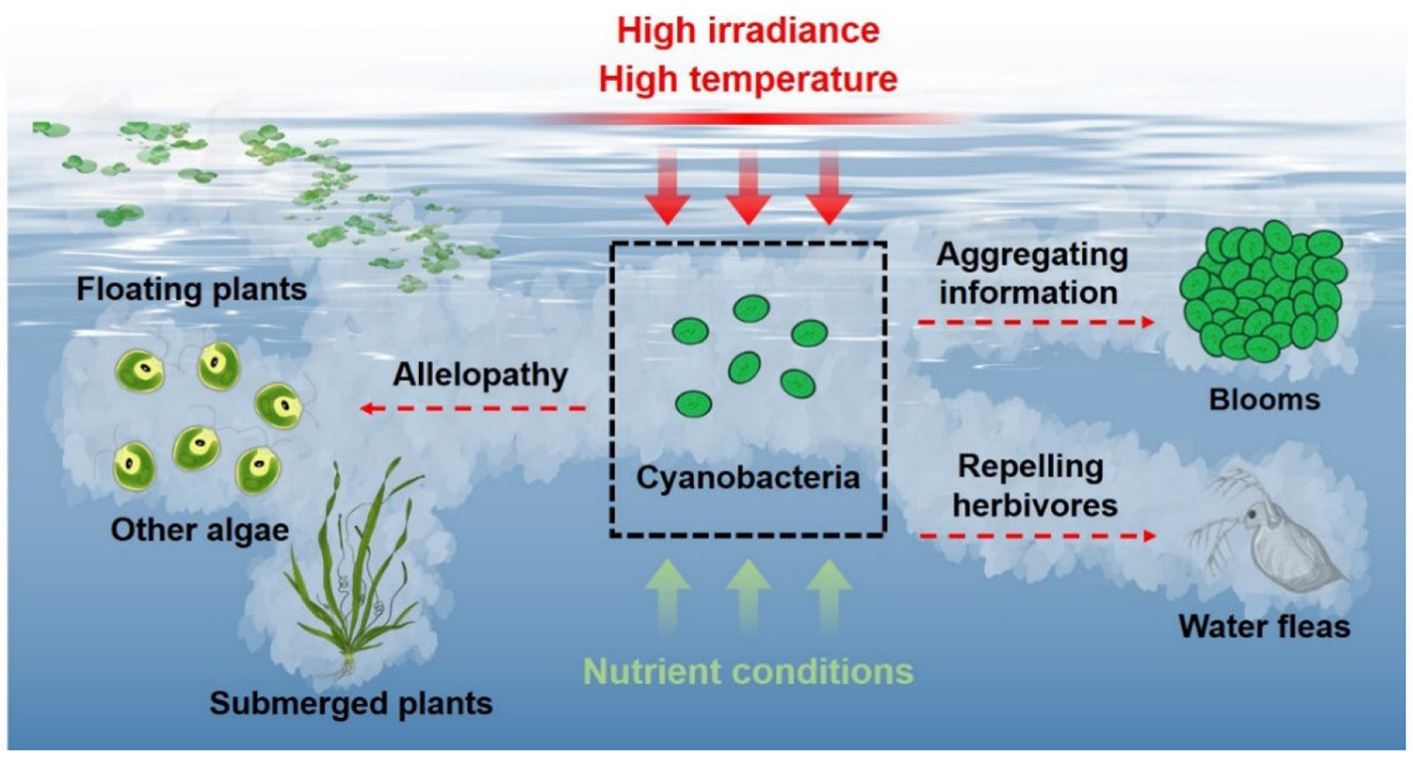
Emission of cyanobacterial VOCs and their roles.

Cyanobacteria easily burst blooms in summer with high irradiance and high temperature, and their massive reproduction and growth can result in relative lack of nutrients. These adverse environmental conditions are not beneficial to cyanobacterial growth (Wang and Li, 2015; Zuo, 2019; Zheng et al., 2020), but promote cyanobacteria releasing a wide spectrum of VOCs by up-regulating related gene expression and oxidatively degrading β-carotene. These VOCs can transfer allelopathic signals to inhibit the growth of algae and aquatic plants, and even directly kill these competitors, which may offset the disadvantageous effects of the environmental conditions and promote cyanobacteria to dominate eutrophicated waters. The VOCs from cyanobacteria and the ruptured cells have repelling function on herbivores, which improves cyanobacterial population survival by reducing the risk of encountering predators. In addition, cyanobacterial VOCs might transfer aggregating information between homogeneous species, then these cells aggregate together (bursting blooms) to resist the adverse conditions (Figure 2). Therefore, cyanobacteria increase the emission of VOCs in response to the adverse environmental conditions, and these VOCs serve important functions in cyanobacterial massive reproduction and bursting blooms.

These findings in cyanobacterial VOCs provide a new thought for recognizing the formation of water blooms, but are still in a primary stage. During water blooms, how do cyanobacteria release VOCs to respond the complex environmental conditions not only a single condition? What is the exact allelopathic mechanism of cyanobacterial VOCs on the competitors? What is the exact repelling mechanism of cyanobacterial VOCs on the herbivores? How are cyanobacterial acceptor cells to identify and sense VOC aggregating information and perform an aggregation response? Among plentiful cyanobacterial VOCs, what is the exact information of each component for different acceptors? These issues should be investigated in future by using protocols in molecular biology, whose answers will promote us to deeply recognize cyanobacteria blooms from VOC perspective for preventing and controlling the blooms.

## Author contributions

The author confirms being the sole contributor of this work and has approved it for publication.
